# The broad impact of cell death genes on the human disease phenome

**DOI:** 10.1038/s41419-024-06632-7

**Published:** 2024-04-08

**Authors:** Abigail L. Rich, Phillip Lin, Eric R. Gamazon, Sandra S. Zinkel

**Affiliations:** 1 Department of Pathology, Microbiology & Immunology, Vanderbilt University Medical Center, Nashville, TN USA; 2 Division of Hematology/Oncology, Department of Medicine, Vanderbilt University Medical Center, Nashville, TN USA; 3 Division of Genetic Medicine, Department of Medicine, Vanderbilt University Medical Center, Nashville, TN USA; 4 Vanderbilt Genetics Institute, Vanderbilt University Medical Center, Nashville, TN USA; 5 Department of Cell and Developmental Biology, Vanderbilt University Medical Center, Nashville, TN USA

**Keywords:** Genetics research, Inflammation

## Abstract

Cell death mediated by genetically defined signaling pathways influences the health and dynamics of all tissues, however the tissue specificity of cell death pathways and the relationships between these pathways and human disease are not well understood. We analyzed the expression profiles of an array of 44 cell death genes involved in apoptosis, necroptosis, and pyroptosis cell death pathways across 49 human tissues from GTEx, to elucidate the landscape of cell death gene expression across human tissues, and the relationship between tissue-specific genetically determined expression and the human phenome. We uncovered unique cell death gene expression profiles across tissue types, suggesting there are physiologically distinct cell death programs in different tissues. Using summary statistics-based transcriptome wide association studies (TWAS) on human traits in the UK Biobank (*n* ~ 500,000), we evaluated 513 traits encompassing ICD-10 defined diagnoses and laboratory-derived traits. Our analysis revealed hundreds of significant (FDR < 0.05) associations between genetically regulated cell death gene expression and an array of human phenotypes encompassing both clinical diagnoses and hematologic parameters, which were independently validated in another large-scale DNA biobank (BioVU) at Vanderbilt University Medical Center (*n* = 94,474) with matching phenotypes. Cell death genes were highly enriched for significant associations with blood traits versus non-cell-death genes, with apoptosis-associated genes enriched for leukocyte and platelet traits. Our findings are also concordant with independently published studies (e.g. associations between *BCL2L11*/BIM expression and platelet & lymphocyte counts). Overall, these results suggest that cell death genes play *distinct* roles in their contribution to human phenotypes, and that cell death genes influence a diverse array of human traits.

## Introduction

Regulated cell death is an essential phenomenon during the development of multicellular organisms [1–3] and dysregulation of cell death is a prominent feature of organismal aging [2, 4]. Three well characterized forms of cell death that rely upon genetically encoded, hierarchical signaling pathways are apoptosis, necroptosis, and pyroptosis [5]. Apoptosis can be elicited via extrinsic or intrinsic cellular perturbation; as a result, there is both “extrinsic” apoptosis regulated by caspases and “intrinsic” apoptosis regulated by BCL-2 family members which regulate mitochondrial membrane permeability [6, 7]. Necroptosis is a regulated form of cellular necrosis which converges on the assembly of an MLKL pore on the cell membrane [8, 9]. Pyroptosis is an immunogenic form of cell death that is canonically reliant upon the formation of the NLRP3 inflammasome and the release of IL-1β and IL-18 via gasdermin membrane pores [10]. Apoptosis, necroptosis, and pyroptosis all ultimately result in cellular demise; however, their mechanisms and functions are unique.

### Three major regulated cell death modalities are implicated in health and disease

Regulated cell death maintains homeostasis in diverse organ systems and dysregulation of cell death has pathophysiological implications [11, 12]. For instance, neurodegenerative disease is associated with inappropriate neuronal cell death and inflammatory cell death pathways [13]. Aberrant upregulation of necroptotic and pyroptotic inflammatory cell death within the bone marrow environment impair hematopoiesis and drives bone marrow failure syndromes [14–16]. Upregulation of pro-survival pathways and the downregulation of pro-death signals are a hallmark of neoplasms [17]; mouse models with both systemic and tissue-specific deficiencies in key apoptotic signaling regulators have illustrated a propensity for the development of malignancy [18–20]. Apoptotic cell death controls proper lymphocyte development and destruction of autoreactive lymphocytes, and defects in apoptotic destruction of these autoreactive lymphocytes result in autoimmune diseases [21]: TNFR-dependent extrinsic apoptosis is crucial for the negative selection of autoreactive thymocytes, and antiapoptotic BCL2 family members play a role in regulation of this process [22–25]; BCL2 family signaling contributes to selection against polyreactive B cells [26–28]. The Mendelian disease autoimmune lymphoproliferative syndrome (ALPS) arises from defects in the genes *FAS*, *CASP8*, and *CASP10*, which coordinate the Fas-dependent apoptosis that is critical for thymocyte development [29–31]. Defects in cell death regulation contribute to a diverse array of pathologies that can be tissue- and context-specific.

### Tissue-specific transcriptional regulation of cell death has implications for human disease

Diverse functions of various tissues dictate distinct cell death behavior [32, 33] despite developing from identical germline DNA. For example, skin cells must be resistant to cell death to perform their barrier function, whereas the dynamic regulation required of the hematopoietic system dictates a similarly adaptable regulation of cell death to maintain homeostasis. This context dependency is established in part by transcriptional regulation of cell death pathway members, which operate downstream of transcription factor programs including Rel/NF-kappaB [34], p53 [35], interferon regulatory factors, among others [10, 36].

Another context-dependent feature of cell death pathway regulation is tissue-specific gene expression patterning. Generally, tissue-specific gene expression patterning is transcription factor *independent* [37] and highly variable across individuals in a population [38, 39]. This variation in expression may explain the incidence of disease across a large population of individuals and/or help us understand disease susceptibility across these populations [40], and is critical for untangling the tissue-specific functions of genes and pathways. Although the role of individual cell death pathway genes has been rigorously defined in mouse models, the specific landscape of the expression of cell death pathway genes across a wide array of non-germline tissues is a fundamental aspect of cell death biology and is heretofore underexplored. Furthermore, the relationship between the expression of cell death genes and human disease susceptibility is not thoroughly understood.

In this study, we characterize the landscape of tissue-specific cell death gene expression across human tissues. From there, we examine the relationship between tissue-specific cell death gene expression and human traits by leveraging summary statistics-based transcriptome wide association studies (TWAS) [41]. This approach uses genome-wide association study (GWAS) summary statistics and reference transcriptomic datasets to identify genetic loci with strong associations between gene expression and a given trait [41, 42]. A summary statistics-based TWAS approach enables us to identify how subtle, lifetime shifts in the predisposition of cell death gene expression associates with clinically relevant traits (e.g. predisposition to disease, lab values, drug susceptibility) across the human phenome, i.e. the complete spectrum of human traits.

## Results

### Curation of an array of core cell death genes

**Fig. 1:**
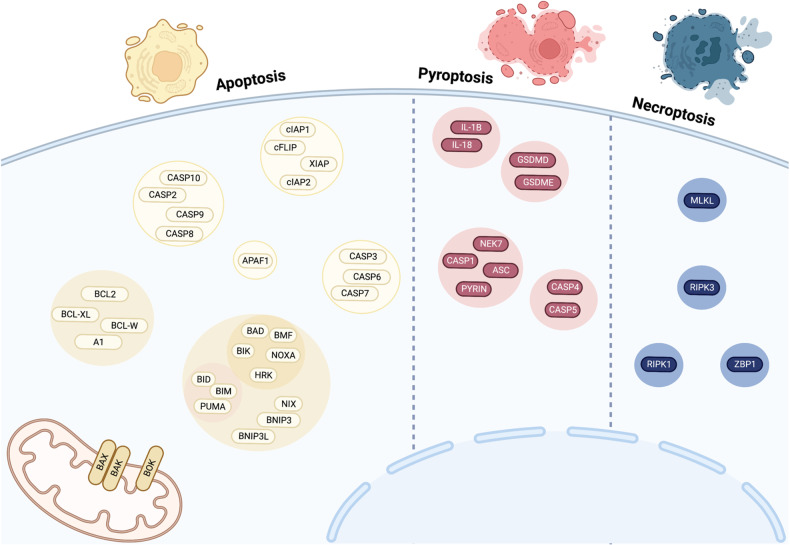
Composition and organization of an array of core cell death genes. Summary of the composition and organization of our chosen cell death gene array by pathway and subpathway: three cell death modalities of interest: apoptosis (yellow), pyroptosis (red), and necroptosis (blue) and the proteins encoded by the genes selected for studying these modalities (ovals). Proteins are grouped by function.

Our studies focus on core programmed cell death machinery in the apoptotic, necroptotic, and pyroptotic pathways that are well studied [43, 44], targetable by multiple clinically relevant pharmaceuticals [45–47], and therefore implicated in the pathogenesis of many diseases [11, 48, 49]. These pathways are comprised of well-defined gene sets that participate primarily in cell death signaling. In all, we chose 44 genes operating within the intrinsic apoptosis pathway (18 genes), extrinsic apoptosis pathway (12 genes), necroptosis pathway (4 genes), and pyroptosis pathway (10 genes) (Fig. 1, Supplementary Table 1). Other biologically relevant cell death pathways, such as ferroptosis [50], cuproptosis [51], and parthanatos [52], are defined by central gene regulators (listed in Supplementary Table 1) that we examined in a separate analysis.

### Observed expression of programmed cell death genes is variable and tissue-specific

**Fig. 2:**
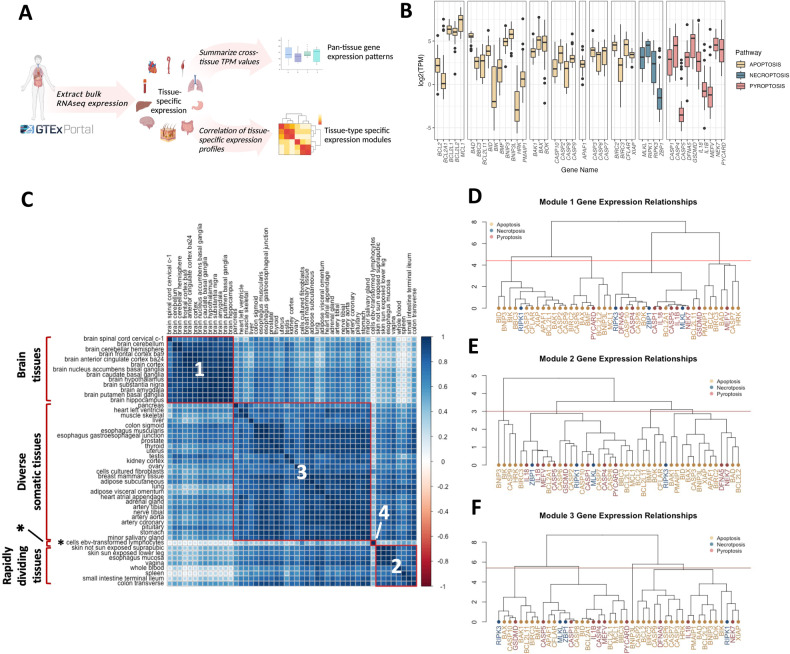
Tissue-specific patterning of cell death gene expression across adult somatic tissues. **A** Preprocessed GTEx tissue expression data in transcripts per million (TPM) from 49 tissues were extracted for analysis, and the distribution of median values in each tissue and the Pearson correlation between median TPM values in each tissue was calculated. **B** Boxplot depicting the distribution of median tissue TPM for GTEx tissues for each cell death gene. Outlier expression values (>1.5X IQR) are depicted as dots. Genes are grouped by pathway. **C** Correlation plot for 49 GTEx tissues using Ward’s D hierarchical clustering reveals four modules (outlined in red) that are highly correlated: brain tissues, rapidly dividing tissues, diverse somatic tissues, and EBV-transformed lymphocytes. **D**–**F**: Dendrograms illustrating relationships between gene expression values in Modules 1 (**D**), 2 (**E**), and 3 (**F**) using hierarchical clustering of Euclidean distance between median gene TPM values across members of expression modules identified in (**C**).

We first examined the expression of members of our cell death gene array across adult non-germline tissues to reveal cell-type-specific patterns in cell death machinery networks (Fig. 2A). We employed the GTEx resource release 8, which has transcriptomic data on tissues for 838 individuals. Across 49 GTEx tissues, expression was highest in genes encoding prosurvival factors *MCL1* (MCL1), *BCL2L1* (BCL-XL), and *BCL2L2* (BCL-W) (Fig. 2B). There was variable expression of all cell death genes evaluated across tissues, which can be visualized by the interquartile range when median log_2_(TPM) expression for each of these genes was examined (Fig. 2B). The variance observed was not explained by the number of expression observations in GTEx, suggesting that these expression distributions are biologically relevant and not the result of sample size (Fig S1A). Two possible interpretations of this variance in cross-tissue gene expression are: expression patterns of cell death genes maintain a similar stoichiometry but differing magnitude across tissues; or expression patterns of cell death genes are variable across tissues. Indeed, we observed that immune-related tissues, whole blood and spleen, featured the highest levels of expression of necroptosis and pyroptosis genes (Fig. 2B, GTEx [38]).

To ascertain if gene expression patterns were maintained across tissues, we computed the correlation of each tissue-tissue pair using expression of all genes in our cell death array. Hierarchical clustering of correlation values subsequently identified four distinct groups of tissues with highly correlated expression of cell death genes (Fig. 2C). Interestingly, these tissues segregated into biological themes as follows: brain tissues, rapidly dividing tissues, diverse non-germline tissues, and transformed lymphocytes (Fig. 2C, Supplementary Table 2). The correlation coefficients derived from examining the relationship between median expression of each cell death gene-gene pair across all tissues in a module ranged widely (from *ρ* = -0.99 to 0.99), indicating that cell death genes can be highly positively correlated or negatively correlated depending on the tissue type examined (Fig. S1A, Supplementary Table 3). These module-specific expression patterns suggest differences in “wiring” of cell death pathways across cell and tissue types. Module-specific gene expression patterning reveals that the most highly related genes transcend classical pathway boundaries (Fig. 2D–F). The distinct patterns we observed highlight the importance of evaluating expression in a tissue- or module-specific context for understanding tissue and organ-specific cell death dynamics. Furthermore, the diverse tissue-specific expression patterns we observed *suggest* that specific human diseases might be associated with tissue-specific changes in cell death gene expression, which we explore in the next section.

### Transcriptome-wide association studies identify associations between genetically determined expression of cell death genes and human disease

#### Joint-Tissue Imputation (JTI) generates prediction models for most cell death genes across human tissues for large-scale use on GWAS summary statistics

**Fig. 3:**
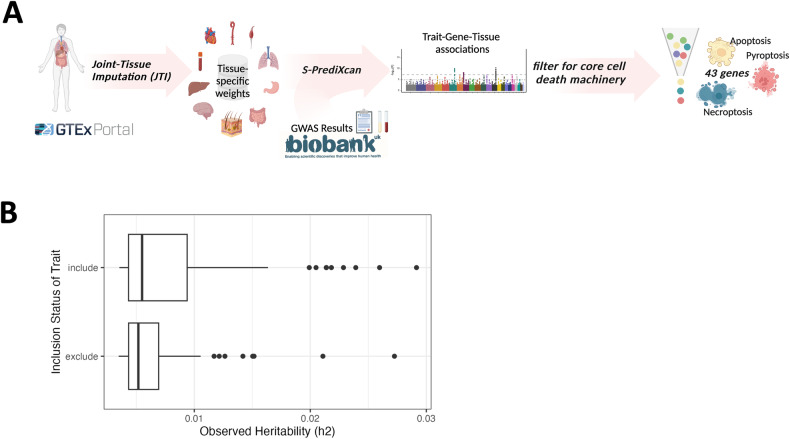
Joint-Tissue Imputation and Biobank TWAS application for phenome-wide scanning of cell death genes. **A** Joint-Tissue Imputation was used to generate tissue-specific weights using the GTEx v8 resource, and these weights were applied to both clinical diagnoses and lab-derived traits from the UKBB, which generated a series of gene-trait-tissue associations that enabled analysis of 43 cell death-associated genes. **B** Observed heritability for ICD10-derived clinical traits that were included or excluded from analysis per our manual curation strategy.

To address the relationship between tissue-specific genetically determined expression of cell death genes and human disease on a tissue-specific level, we implemented a summary statistics transcriptome-wide association study (TWAS) approach, Joint-Tissue Imputation (JTI) methodology [53], on 49 separate GTEx tissues (Fig. 3A, Supplementary Table 4). Joint-tissue imputation generated in silico genetic variation-based models of gene expression for 43 autosomal genes of our array of 44 cell death genes involved in apoptosis, necroptosis, and pyroptosis. Two genes, *CASP7* and *BAK1*, had sufficient QTL information (i.e., gene expression heritability, or the level of genetic control) for testing in all 49 GTEx tissues, whereas *BCL2A1* was included in two tissue-models (Supplementary Table 5, Fig. S2A). *XIAP*, an X-chromosomal gene, was modeled in 19 tissues (Supplementary Table 4). This variability in tissue modeling suggests that cell death genes may have variable level of genetic control of expression across tissues. These JTI models can then be applied to GWAS summary statistics to estimate the association between genetically-determined gene expression and human traits. Because the level of genetic control varies with tissue, certain genes are tested more frequently for each trait than others in these analyses, and some gene/tissue relationships are not tested. At least 10 genes were tested in each tissue (Fig. S2B). Overall, these models test for associations between 1061 cell death gene/tissue pairs and any trait of interest for which GWAS summary statistics are available.

#### Manual curation of relevant clinical outcome and lab-derived traits from the UKBB enriches for heritable traits

To maximize the power of our analysis and enhance the interpretability of findings, we manually curated a list of clinically relevant traits from the UKBB version 3 release (see Data Availability) on which we performed analyses on autosomal genes. This curation prioritized quantitative traits from lab-derived tests and electronic health record (EHR)-derived clinical outcomes with clear diagnoses, specific etiology, and/or minimal redundancy with other UKBB GWAS entries. Ultimately, we selected 482 EHR-derived clinical outcome traits (“clinical outcome traits”) from the UKBB and 31 lab-derived clinical traits from quantitative blood and urine measurements for analysis (“lab-derived traits”) (Supplementary Table 6). The clinical outcome traits span 16 discrete phenotypic categories, and the 24 lab-derived traits encompass both blood and urine-derived markers (Supplementary Table 6). The average heritability of selected clinical outcome traits (as determined by linkage disequilibrium score regression; Data Availability) was higher than that of omitted clinical outcome traits (Fig. 3B). This suggests that our filtering strategy not only enriches for traits with clinical relevance, but traits for which these genetically informed analyses are more relevant. Of these curated clinical outcome traits, 106 phecode-defined traits were available for de novo X-chromosomal *XIAP* association analysis (Supplementary Table 7).

The continuous lab-derived traits and binary clinical outcome traits had marked differences in sample size and the number of significant associations (Fig. S2D). As such, lab values were analyzed separately from clinical traits. Associations from the 24 lab-derived traits were analyzed with an FDR cutoff of 0.01. Given the phenome-wide scope of the clinical outcome trait analysis and subsequent multiple testing burden, to maximize the discovery potential for clinical outcome trait associations, we used an FDR cutoff of 0.25 (corresponding with an unadjusted *p*-value 7.7e-5).

### Genetically determined expression of cell death genes associates with health-related traits across the human phenome, and is unique across specific genes and traits

For clinical outcome traits, we identified 157 significant associations (FDR < 0.25) in 21 cell death genes across 27 unique traits and 12 phenotype categories within our autosomal gene analysis and no significant (FDR < 0.05) associations for X-chromosomal analysis (Supplementary Table 8). Significant associations were detected across all 49 examined tissues (Supplementary Table 8). The number of significant associations identified for a given cell death gene/tissue pairing was not correlated with the number of tissue samples used to generate the tissue-specific models (i.e., weights), suggesting that tissue biology rather than sample size drives the number of associations (Fig. S2A). Similarly, the number of significant associations detected was not correlated with the number of cases used to perform association studies on clinical traits, suggesting that these analyses can detect bona fide biological signals (Fig. S2D). Though tissue specificity is an important biological consideration, we found that for gene/trait associations in multiple tissues where *p* < 0.05, the overwhelming majority of associations were concordant in their direction of effect (Supplementary Table 8). This phenomenon implies that, for traits with multiple tissue associations, there is a shared genetic architecture that drives associations across tissues.

**Fig. 4:**
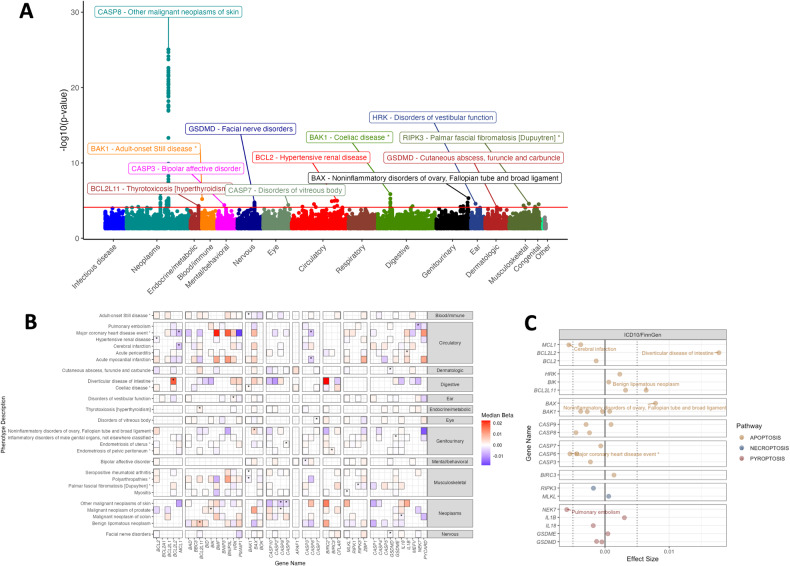
UKBB clinical diagnoses associated with genetically regulated expression of cell death genes identified by S-PrediXcan. **A** Manhattan plot illustrating top gene-trait associations by *p*-value and organized by trait type. Only the most significant gene-trait association in a phenotype category is labeled, and the red line illustrates the FDR = 0.25 threshold (*p* < 7.7e-5). Gene-trait associations with significant associations across multiple tissues (i.e. *CASP8* and Other malignant neoplasms of skin), are not annotated for clarity. **B** Heatplot illustrating median effect size and direction across gene-trait-tissue associations for traits with n≥1 associations with FDR < 0.25 (labeled with “*”) or *p* < 0.05. **C** Median effect size for significant (FDR < 0.25) gene/trait/tissue associations where associations with |*β*| > 0.005 are labeled.

We observed that genetically determined expression of a suite of cell death genes is associated with a range of clinically relevant traits comprising over a dozen different phenotypic categories (Fig. 4A). The most highly significant association was between *CASP8* expression and “Other malignant neoplasms of skin”. *CASP8* associations could be detected in a variety of different tissues (Supplementary Table 8, Fig. 4A). This suggests that there are tissue-shared eQTLs for the gene driving the associations across tissues. Significant associations were observed in genes involved in all examined cell death pathways, involving apoptotic, necroptotic, and pyroptotic genes (Fig. 4A). These findings reveal a potential link between genetically determined expression of apoptosis, necroptosis, and pyroptosis machinery in the etiology of a variety of clinically relevant disorders.

A key feature of the gene-level TWAS approach is its ability to estimate both effect size and direction of effect for associations. Given the conserved structural and functional characteristics of many of the genes in our cell death array, we sought to resolve significant gene-trait association effect sizes by both gene similarity and trait similarity (Fig. 4B). We were surprised that more significant associations did not occur in groups of genes with functional similarities (e.g., Initiator Caspases, prosurvival BCL-2 family genes, as grouped on the X-axis in Fig. 4B). To enrich for potential additional relevant patterns, we graphed median effect sizes for nominally significant (*p*_unadj_<0.05) gene/trait associations alongside the significant gene/trait associations (as denoted by asterisks, Fig. 4B). The effect size and direction of shared associations for a particular trait was variable (Fig. 4B). Across significant (FDR < 0.25) gene/trait associations, the largest magnitude of effect was the association of diverticular disease with lower expression of *BCL2L2*, encoding BCL-W (Fig. 4C). Explicitly, this means that the genetically determined expression of BCL-W accounts for almost 3% of the genetic predisposition to diverticular disease of the intestine. These results provide preliminary evidence there is a directional relationship between cell death gene expression and a suite of human traits and support a model in which functionally similar genes and pathways have distinct roles in the etiology of disease.

### Laboratory traits are enriched for significant associations with hematologic phenotypes

**Fig. 5:**
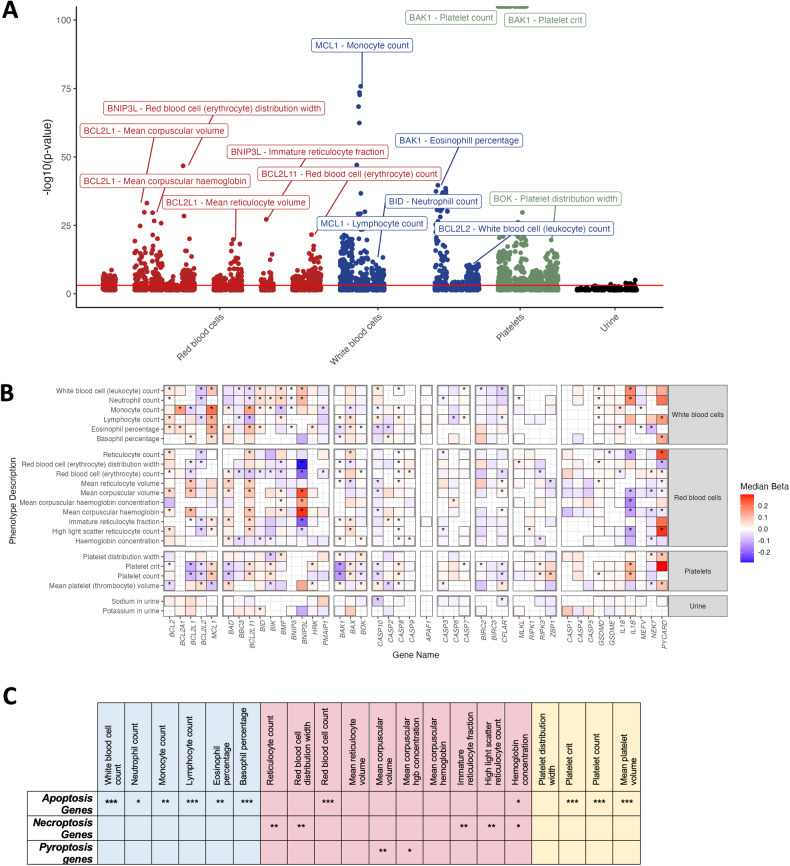
Lab-derived blood & urine metabolite traits associated with genetically regulated expression of cell death genes identified by S-PrediXcan. **A** Manhattan plot illustrating top laboratory-derived gene-trait associations by *p*-value and organized by trait type. Unique trait associations with *p*_unadj_ < 2e-10 are labeled, and the red line illustrates the FDR = 0.01 threshold (*p* < 0.0008353). Gene/trait associations across multiple tissues are omitted from this plot for clarity. **B** Median effect size and direction across all gene/trait pairs with a tissue association of *p* < 0.05. Significant (FDR < 0.01) gene/trait associations are indicated by an asterisk. **C** Fisher’s Exact testing for enrichment of associations for apoptosis, necroptosis, and pyroptosis genes as defined in Table 1. *p < 0.05, **p < 0.01, ***p < 5e-10.

We identified hundreds of significant (FDR < 0.01) gene/tissue/trait associations for lab-derived traits. The most highly significant associations were in blood traits, particularly associations between *BAK1* and platelet count & crit (Supplementary Table 9). Other highly significant associations were observed between leukocyte blood parameters and *MCL1*, and *BCL2L1* (encoding BCL-XL) and red blood cell parameters (Fig. 5A). As was observed in our analysis of EHR-derived traits, the direction of effect for gene/trait/tissue associations across tissues were overwhelmingly concordant for any given trait.

Resolving significant (FDR < 0.01) and nominally significant (*p* < 0.05) association effect sizes by gene family and trait family reveal a matrix of gene/trait associations that are not redundant across similar gene families (Fig. 5B; e.g., *BCL2L1* encoding BCL-XL and *MCL1* display associations with opposite directions of effect across blood traits). The maximum associations by median effect size were observed in red blood cell-associated traits and with BH3-only gene *BNIP3L* and pyroptosis-associated adapter *PYCARD* (Fig. 5B). For many of these associations, up to 20% of the genetic contribution for several traits can be traced back to genetically determined expression of one of these two genes (Fig. 5B). On the whole, these findings reinforce the proposition there are distinct roles for cell death genes that are considered functionally redundant in their contribution to human phenotypes and indicate that the genetically determined expression of *BNIP3L* and *PYCARD* exert a significant effect on hematologic, particularly red blood cell traits.

There were many more highly significant associations with large-magnitude effect sizes for hematologic traits relative to urine traits (Fig. 5A, B), suggesting that cell death gene expression is uniquely important for shaping hematopoiesis. To explicitly test if cell death pathway genes were overrepresented among our significant associations, we performed enrichment analysis on apoptotic, necroptotic, or pyroptotic gene sets across each of the blood traits. Cell death genes were highly enriched for significant associations with blood traits versus non-cell-death genes, with apoptosis gene sets enriched for significant associations with leukocyte and platelet traits, and necroptosis and pyroptosis gene sets enriched for associations with erythroid traits (Fig. 5C). Overall, these results suggest that the genetically determined expression levels of cell death genes are particularly important in shaping the numbers and distributions of blood and immune cells.

We performed TWAS of clinical outcome and lab-derived phenotypes from the central regulators of ferroptosis, cuproptosis, and parthanatos and identified strong associations between *PARP1* and neoplasms (Fig. S3A) and platelet traits (Fig. S3B). Associations arising from *GPX4* include nasal polyps (Supplementary Table 10). These results align with PARP1’s known role as a DNA damage response coordinator [52] and reports of ferroptosis’ role in the pathogenesis of nasal polyps [54].

### Biobank- and literature-based replication of gene/trait associations

**Fig. 6:**
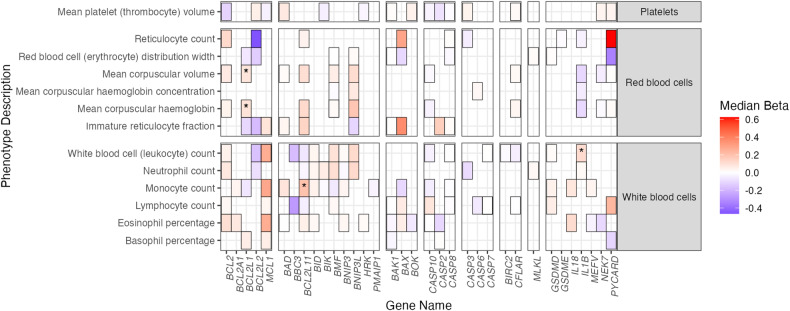
External validation of blood traits in the BioVU biobank. Median effect size and direction of gene/trait TWAS associations from the BioVU dataset that replicated UKBB analysis associations. Concordant associations that are significant in the UKBB (FDR < 0.01) and BioVU (*p* < 0.05) are boxed. Concordant gene/trait associations surpassing a more stringent multiple testing threshold within BioVU (FDR < 0.05) are indicated by an asterisk.

Our discovery analysis implicated genetically determined expression of cell death genes in dozens of clinically relevant diagnoses that are viable candidates for validation. We opted for a two-pronged approach that [1] replicated our in silico results in an independent, large-scale biobank and [2] identified in vivo and in vitro studies that are concordant with our findings via an extensive literature review. We performed external replication analysis by applying our TWAS methodology to a large-scale DNA biobank linked to electronic health records at Vanderbilt University Medical Center, BioVU (*n* = 94,474 individuals of predominantly European ancestry) [55]. For clinical outcome traits, we prioritized top gene/trait associations that had corresponding phecodes in the BioVU dataset. Remarkably, we detected significant associations between *BAK1* and Rheumatoid arthritis/Polyarthropathies (*p* = 2.50e-3) as well as *BCL2L2* and Diverticular disease of the intestine (*p* = 3.86e-02) (Table 1). For lab-derived traits, we performed replication analyses on 13 available hematologic parameters (Fig. 6). Dozens of associations, for instance *BCL2L11*/BIM and monocyte count, aligned with findings from our discovery analysis (Fig. 5B).

**Table 1:** Independent replication analysis in the BioVU biobank.

Phecode	Effect	*p*-value	Gene	Phenotype	# cases	# controls
714	−0.282262	2.50E-03	*BAK1*	Rheumatoid arthritis and other inflammatory polyarthropathies	928	19052
562.2	0.800272	3.86E-02	*BCL2L2*	Diverticulitis	291	16064

We further evaluated concordance of our findings with observations from murine knockout models (representing very low/no gene expression) and reports from hypothesis-driven human genetic studies may lend support to our observations as well. Table 2 compares traits reported in independent, peer-reviewed publications with those identified in our PheWAS. These studies support our findings for 19 measurable phenotypes in highly controlled experimental systems. One such example is that of *BCL2* and its associations with both lymphocyte count, and hypertensive renal disease, as murine knockout of *Bcl2* reports lymphoid and kidney development effects (Table 2, [56]). Overall, our replication analysis and literature review provide two independent lines of evidence that support the utility of our approach to discovering novel gene/trait associations.

**Table 2:** External validation studies for top blood trait associations (gene/trait associations in >10 tissues; FDR < 0.01) and top health trait associations (FDR < 0.25).

Gene	PheWAS Associations (direction of effect)	Mouse Studies	Human Studies
*BAD*	Platelet count (−)	Kelly et al. [66]. *Bad*−/− mice have elevated platelets.	
*BCL2L11*	White blood cell count (−), platelet count (+), lymphocyte count (−)	Bouillet et al. [67] *Bim*−/− mice have increases in peripheral blood WBCs, lymphocytes, monocytes, and granulocytes with a reduction in platelets.	
*BNIP3L*	High light scatter reticulocyte percentage (+/−), immature reticulocyte fraction (+/−)	Sandoval et al. [68]. *Bnip3l*−/− mice have reticulocytosis and anemia	
*BCL2*	Lymphocyte count (+)	Veis et al. [56]. *Bcl2*−/− mice exhibit lymphoid tissue involution with age.	
*BCL2L1*	Red blood cell count (+/−)	Motoyama et al. [69]. *Bcl2l1*−/− embryonic stem cells have defects in late stage erythropoiesis and do not contribute to definitive erythropoiesis. Rhodes et al. [58]. Bcl-x(L) prevents late-stage erythroblast apoptosis.	
*MCL1*	Lymphocyte count (+)	Opferman et al. [70]. Conditional *Mcl1* deficiency in lymphocytes (*Lck*Cre, *Cd19*Cre) ablates T & B lymphocytes.	
*CASP8*	Red blood cell count (−); lymphocyte count (−)	Varfolomeev et al. [71]. *Casp8*−/− embryos exhibit profound erythrocytosis. Salmena & Hakem. [72]. T-cell specific deletion of *Casp8* results in an age-dependent lymphoproliferative disorder.	Grzela et al. [73]. Low *CASP8* expression is a feature of lymphocytes derived from a patient with human autoimmune-like lymphoproliferative syndrome
*CASP10*	Lymphocyte count (+/−)	No direct murine paralog.	Wang et al. [74]. Missense mutations in *CASP10* associate with human autoimmune lymphoproliferative syndrome and defects in lymphocyte apoptosis.
*BCL2L11*	Benign lipomatous neoplasm (+), thyrotoxicosis (+)	Bouillet et al. [67]. *Bim*−/− mice develop plasmacytosis; autoimmune kidney disease.	
*BIK*	Malignant neoplasm of prostate (+/−)		Wang et al. [75]. Novel risk variants for prostate cancer identified in the *BIK* locus in multi-ancestry GWAS.
*HRK*	Disorders of vestibular function (+)	Coultas et al. [76]. *Hrk* deficiency protects sensory neurons from nerve growth factor deprivation.	
*BAX*	Noninflammatory disorders of ovary, Fallopian tube and broad ligament (+)	Knudson et al. [77]. *Bax*−/− mice exhibit ovarian aberrations.	
*BAK1*	Celiac disease (+), adult-onset Still disease (−), polyarthopathies (−), seropositive rheumatoid arthritis (−)		Chernavsky et al. [78]. *BAK1* mRNA is increased 2-fold in Celiac disease patients.
*BCL2*	Hypertensive renal disease (−)	Veis et al. [56]. *Bcl2*−/− mice exhibit defects in renal development.	
*BCL2L2*	Diverticular disease of intestine (+)	Pritchard et al. [79]. Bclw deficiency enhances intestinal apoptosis in response to cytotoxic insult.	
*CASP7*	Disorders of vitreous body (−)	Choudhury et al. [80]. *Casp7*−/− mice are resistant to loss of retinal ganglion cells following optic nerve injury.	
*GSDMD*	Cutaneous abscess, furuncle and carbuncle (−)	Liu et al. [81]. *Gsdmd*−/− mice develop larger skin abscesses when infected with *S. aureus*.	
*IL1B*	Acute pericarditis (+)		Thorolfsdottir et al. [82]. Genetic variants influencing *IL1* transcription associate with pericarditis.
*IL18*	Malignant neoplasm of colon (−)	Salcedo et al. [83]. *Il18*−/− mice are more susceptible to colitis (AOM/DSS model)-induced polyp formation.	

## Discussion

This study presents a comprehensive transcriptomic survey of cross-tissue cell death gene expression and delivers an atlas of human traits that are influenced by genetically determined expression of apoptotic, necroptotic, and pyroptotic genes. Our analysis identified dozens of human phenotypes that are associated with cell death gene expression, both novel and previously reported. Many of these phenotypes were dually validated in an independent biobank (Table 1 & Fig. 6) and have been reported as part of independent, peer-reviewed publications (Table 2). Beyond identifying novel relationships between cell death genes and human phenotypes, our findings highlight several important phenomena.

TWAS analyses such as those that form the basis of our PheWAS have utility in their ability to identify relationships between extremes in gene expression and traits. This transcends existing protein structure/function paradigms in the cell death field. Though associations do not imply causal relationships (i.e., lower expression of *GeneX* causes *DiseaseY*), the findings may be of utility in the vetting and application of cell death inhibitory compound administration, as the outcome of restricted gene expression is lower protein content/function. We observed that low expression of *BCL2L1* (encoding BCL-XL) is significantly associated with a decrease in platelet count and platelet crit (Fig. 5B). Correspondingly, the major dose-limiting toxicity of an inhibitor of BCL-XL, navitoclax, is its reduction in platelet count [57]. Many of the tested genes have associations with multiple hematopoietic traits, highlighting the highly interdependent nature of hematopoietic cell differentiation and the importance of cell death for hematopoiesis. For instance, increased expression of *BCL2L1* is strongly positively correlated with mean corpuscular volume (MCV) and mean reticulocyte volume (Fig. 5B). This is consistent with observations that BCL-XL deficiency impairs late erythroblast/reticulocyte survival [58].

An intriguing phenomenon across functionally similar gene groupings (defined in Fig. 1) was the presence of significant gene/trait associations with opposite directions of effect. For example, *BAX* and *BAK1* are thought to be functionally redundant, as dual knockout of these pore-forming proteins is required to elicit multi-organ pathologies [59]. *BAK1* and *BAX* had divergent median effect sizes when considering platelet count/crit, eosinophil percentage, and mean reticulocyte volume (Fig. 5B). Furthermore, *BCL2L1* and *MCL1*, both prosurvival proteins, had discordant associations with platelet count & platelet crit, monocyte count, and immature reticulocyte fraction (Fig. 5B). These results highlight the potential for non-redundant roles of apoptotic family genes in human hematologic traits.

Though there are gene-specific patterns across all gene/trait associations from our analyses, overall, cell death gene expression associates with many hematologic traits. Cell death genes were highly enriched for significant associations with blood traits versus non-cell-death genes, with apoptosis-associated genes enriched for leukocyte and platelet traits and necroptosis gene associations enriched for erythroid traits (Fig. 5C). This reinforces the paradigm that apoptosis pathway genes are critical for white blood cell and platelet development and suggests that immunogenic/proinflammatory cell death pathways play an important role in regulating erythropoiesis.

Our cross-tissue survey of expression patterns revealed discrete tissue modules with correlated gene expression signatures (Fig. 2C). Using only our array of 44 genes, we identified biologically coherent tissue modules that segregated into relevant groupings: nervous tissues (module 1), which are comprised largely of a pool of post-mitotic cells, segregated clearly from a group of rapidly dividing tissues including intestines, whole blood, and skin (module 3), and these were separated by an intermediate module [2] with various somatic tissues. Surprisingly, the most highly correlated gene pairs within each module (Fig. 2D-F) were not gene paralogs, nor did many highly correlated genes reside in the same pathways, suggesting that cell death gene expression networks have unique architecture in specific tissues (Supplemental Table 3). Notably, EBV-transformed lymphocytes comprised a module distinct from all primary tissues examined (Fig. 2C), suggesting that this cell type is divergent from other tissue types with regards to cell death gene expression. The unique transcriptional signature in these immortalized cells highlights a limitation of using transformed lymphocytes in studies in which the dynamics of cell death are important for the readout (e.g., drug toxicity screens, MPRAs). Indeed, the EBV-encoded E1B protein functionally complements BCL-2 in its anti-apoptotic action to facilitate lymphocyte transformation [60]. As such, these findings advocate for careful selection of mechanistic models that avoid transformed cell lines and recapitulate the transcriptional “footprint” of the target tissue or organ system.

This study has limitations that must be considered in the interpretation of our findings. The TWAS methodology models how germline, rather than somatically acquired, genetic variants influence gene-level expression, rather than protein-level expression, leveraging expression quantitative trait loci (eQTLs) [40]. As such, this study captures how germline-mediated differences in gene expression across a lifetime influence traits. Rare gain- or loss-of function mutations in our cell death gene array, that may have a substantial impact on the functioning of these pathways, are also not captured in this study. Such variants have been captured by Karczewski and colleagues (2022) [61], who identified germline rare variants in cell death genes that associate with human traits via whole exome sequencing of large populations. Current data in large-scale biobanks preclude comparative analysis of gene expression/protein abundance associations, however, there is significant sharing of regulatory information between gene and protein-level expression QTLs [62, 63], suggesting that our results may extrapolate to protein-level phenomena. Databases for interrogating pathogenic mutations of cell death genes and protein structures/post-translational modifications are described more deeply elsewhere [64]. Our transcriptome training models were derived from the sampled individuals in GTEx v8, and the GWAS summary statistics data were derived from the UKBB, resources that are biased towards individuals of European ancestry, limiting our ability to make cross-ancestry generalizations. Power to detect associations is influenced by the specificity of diagnosis codes within the UKBB and the number of cases per trait (for instance “Other malignant neoplasms of skin” diagnosis code may encompass a range of distinct etiologies, and autoimmune lymphoproliferative syndrome was not amongst the traits we analyzed).

Our study identified and replicated novel associations between genetically determined expression of cell death genes and human traits and defined hundreds of significant (FDR < 0.01) associations with lab derived traits. Overall, these results suggest that the genetically determined expression of cell death genes is particularly important in shaping the numbers and distributions of blood and immune cells. Our findings reinforce the proposition there are *distinct* roles for cell death genes that are classically considered functionally redundant in their contribution to human phenotypes. These associations have implications for personalized medicine including disease risk prediction, pharmaceutical candidate screening, and diagnostics for a variety of traits. Ultimately, these findings emphasize the nuance of cell death gene regulation and underscore the importance of cell death pathways in the determination of traits across the phenome.

## Methods

### Parameters for selection of cell death gene array

We focused on apoptotic, necroptotic and pyroptotic signaling pathways to conscribe the bounds of the analysis. Intrinsic and extrinsic apoptosis, necroptosis, and pyroptosis are examples of highly studied and well-defined pathways whose genetically encoded machinery participates primarily in the process of cell death, with minimal shunting to metabolic pathways. Omitted were cell surface receptors initiating these pathways, enzymes involved in non-core post-translational modifications of pathway members, and pathway proteins that participate in but operate on the periphery of these pathways. Omitted also were genes/pathways defined in part by genes with secondary functions (for instance, glutamate transporters *SLC7A11* and *SLC3A2*, which modulate glutamate substrate availability upstream of GSH/GPX4 for ferroptosis, play a significant role in metabolism and other cell processes [50]).

### Gene expression correlation and clustering analyses

Preprocessed GTEx v8 tissue expression data in transcripts per million (TPM) from 49 tissues were extracted for analysis. Median TPM values for each gene in the of the cell death gene array were calculated each tissue, and then applied to Pearson correlation analysis. Ward’s D hierarchical clustering was implemented to identify four discrete tissue modules by graphing the correlation coefficients using the ‘corrplot‘ v0.92 package in R 4.2.1. Euclidean distance between median gene expression TPMs across modules was applied to generate dendrograms for gene-gene relationships across modules.

### Manual curation of health-related outcome traits

To enrich our results for clinically informative traits, we considered health-related outcome phenotypes derived from ICD10 Diagnosis codes (UK Biobank Data Field 41270) as well as multi-parameter phenotypes derived in collaboration with the FinnGen consortium (“FinnGen custom” phenotypes) from the UKBB GWAS round 2 analysis (see Supplementary Data). These encompass 1144 unique phenotypes (Supplementary Table 6) with varying degrees of overlap. ICD10 codes categorized as representing “Pregnancy, childbirth, and the pueperium” (Chapter XV / “O” prefix), “Symptoms, signs and abnormal clinical and laboratory findings, not otherwise classified” (Chapter XVIII / “R” prefix), “Injury poisoning and certain other consequences of external causes” (Chapter XIX / “S” and “T” prefixes), and “Factors influencing health status and contact with health services” (Chapter XXI / “Z” prefixes) were excluded from analysis, as they generally have lower genetic heritability as estimated by linkage disequilibrium score regression (Fig. 3B). GWAS summary statistics calculated after sex stratification were omitted from our analysis as well. Additional manual review of these traits removed FinnGen custom phenotypes that overlapped with more specifically defined or identical ICD10 Diagnosis Codes as well as phenotypes representing nonspecific or “catch-all” traits (Supplementary Data).

Laboratory-derived traits with measurements derived from quantitative assays were selected for analysis, and GWAS summary statistics that were calculated using inverse rank-based normal transformation (IRNT) were chosen for final analysis. This resulted in 31 continuous traits derived from blood and urine tests. Of these traits, some measurements derived from complete blood count data were redundant and removed. These were: monocyte percentage, lymphocyte percentage, neutrophil percentage, high light scatter reticulocyte percentage, reticulocyte percentage, and mean sphered cell volume.

### Joint-Tissue Imputation (JTI) and Transcriptome-Wide Association Study (TWAS)

The summary statistic-based S-PrediXcan methodology developed by Barbeira et al. [41] was employed using tissue-specific models generated using the Joint-Tissue Imputation (JTI) methodology developed by Zhou et al. [53].

Overall, 1061 gene/tissue pairs x 513 traits were assessed, resulting in 544,293 individual tests. The Benjamini-Hochberg multiple hypothesis testing correction was employed to adjust for this large multiple testing burden. A false-discovery rate cutoff of 25% was chosen for clinical outcomes traits to enable a trait discovery-based analysis. Given the higher sample size and sensitivity of continuous trait analysis, we opted to use a more stringent FDR cutoff of 1% for data presentation and enrichment analysis.

### Replication analysis in BioVU

We performed replication testing of our most significant findings (Table 1 and Supplementary Table 11) in an independent biobank linked to electronic health record data, Vanderbilt’s BioVU repository. We tested *BAK1*’s association with Rheumatoid arthritis and with polyarthropathies, *BCL2L2*’s association with Diverticulitis, and *MCL1*’s association with cerebral infarction BioVU using phecodes aligning with ICD10 codes used within the discovery analysis. We were unable to test for replication of *CASP8*’s association with “Other malignant neoplasms of skin,” our most significant association, due to lack of a single phenotype designation in the replication dataset. Replication of blood traits was performed using the JTI/TWAS methodology described above using GWAS summary statistics derived from 94,474 individuals released previously [65].

### Supplementary information

Supplementary Tables

Supplementary Figures

## Data Availability

JTI was performed using GTEx v8 transcriptomic data (dbGaP phs000424.vN.pN). GWAS summary statistics and heritability data were obtained from the publicly-available UKBB v3 release at http://www.nealelab.is/uk-biobank and https://nealelab.github.io/UKBB_ldsc/downloads.html, respectively. Source code and implementation notes for S-PrediXcan and JTI, as well as custom code for figure generation and analyses can be found in this GitHub repository (https://github.com/gamazonlab/CellDeathOmics/).
