# Supplementary material for: The broad impact of cell death genes on the human disease phenome
Source: Cell Death Dis. 2024 Apr 8;15(4):251. doi: 10.1038/s41419-024-06632-7 (PMC11002008; doi:10.1038/s41419-024-06632-7)
Supplement: Supplementary file 2 — Supplementary Figures [file 41419_2024_6632_MOESM2_ESM.pdf]

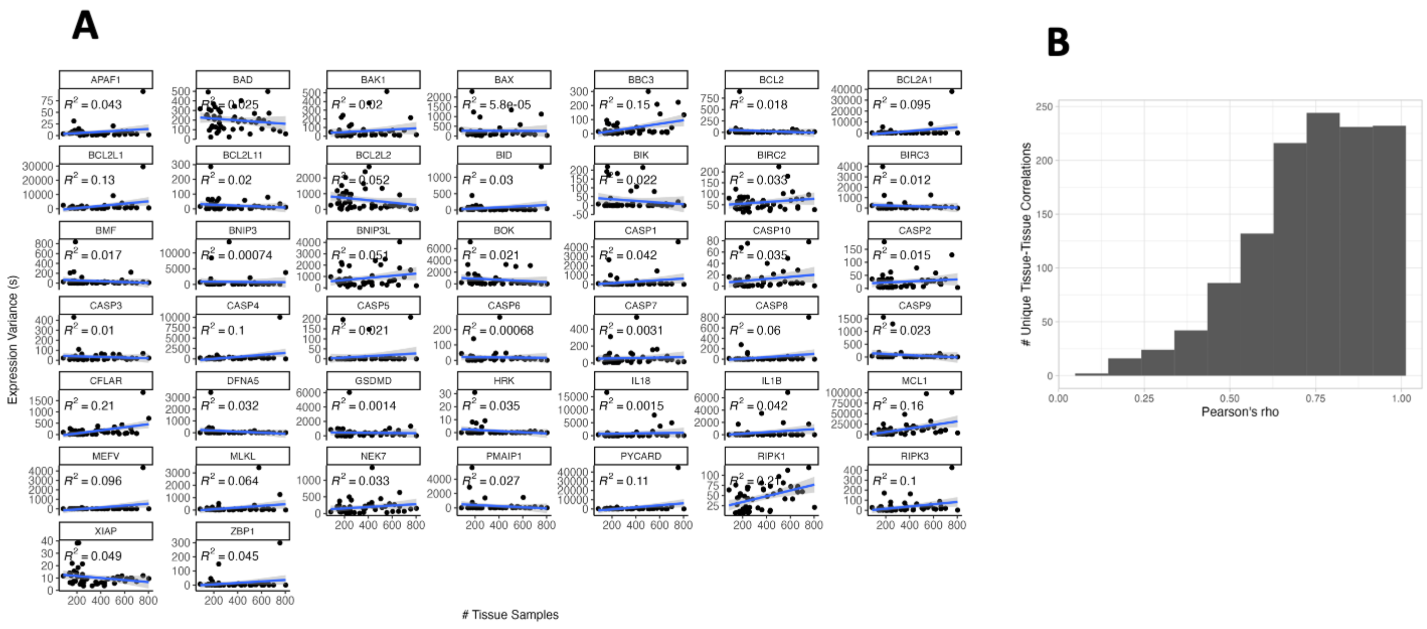


**Supplementary Figure 1: Observed cell death gene expression correlations.**

**A** Linear relationship between the number of GTEx v8 samples with gene expression data for a tissue and the variance in the sample's gene expression values (in TPM) across the cell death genes examined.

**B** Histogram depicting the frequency of tissue-tissue correlation values arising from correlation analysis of median TPM of cell death gene array transcripts in GTEx tissues.

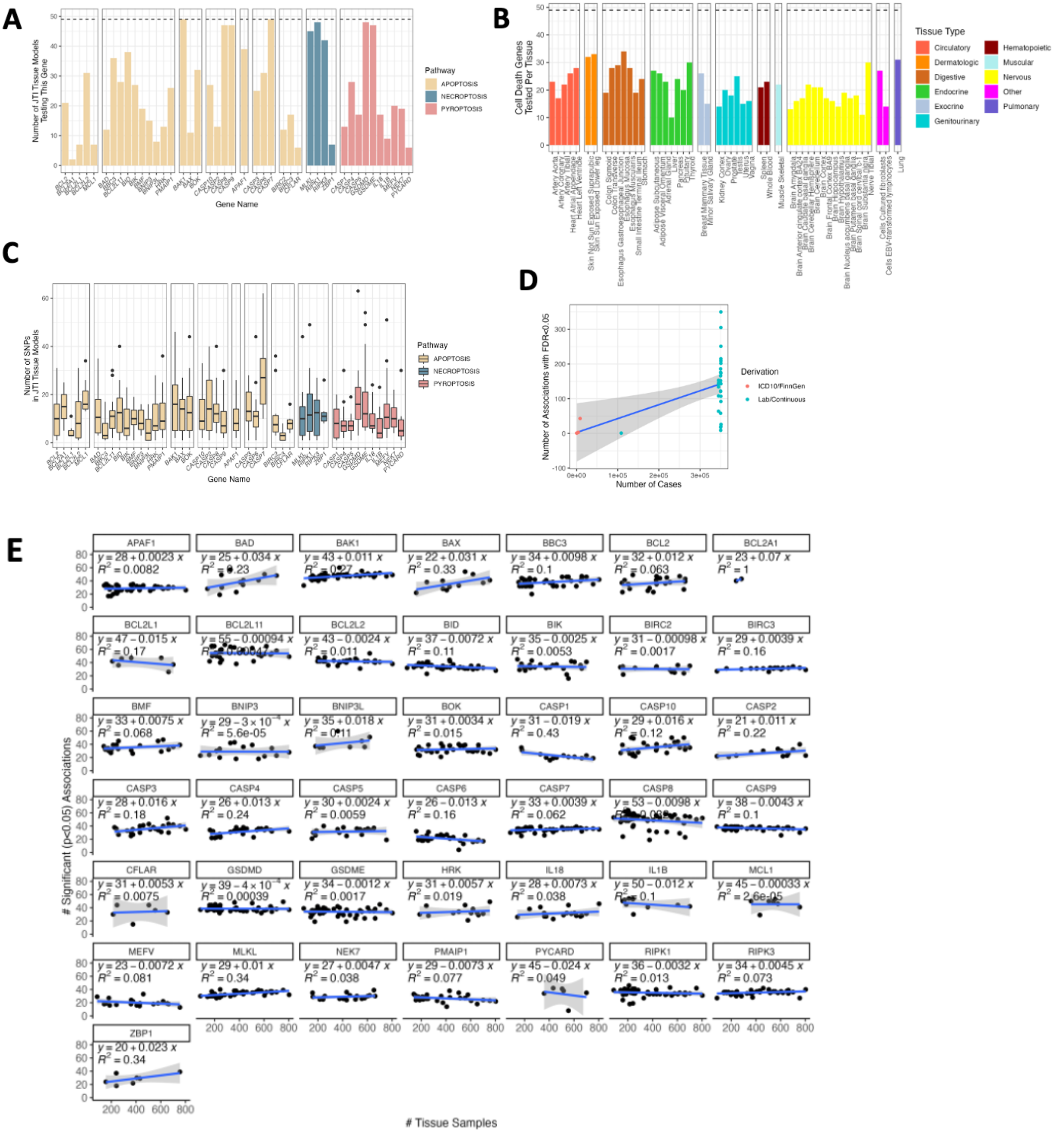


**Supplementary Figure 2: Joint Tissue Imputation Modeling and phenome wide scan results.**

**A** Number of tissues for which cell death genes were included in JTI association testing weights varies by gene.

**B** Number of cell death genes tested in each tissue.

**C** Distribution, across all tissues for which a given cell death gene is modeled, of the number of SNPs (eQTLs) included in JTI models.

**D** Relationship between the number of cases available for significant traits and the number of associations with FDR<0.05.

**E** For each gene in the cell death array, the correlation between the number of GTEx samples used to generate weights for each tissue and the number of significant ($p<0.05$) associations identified in that gene/tissue combination.

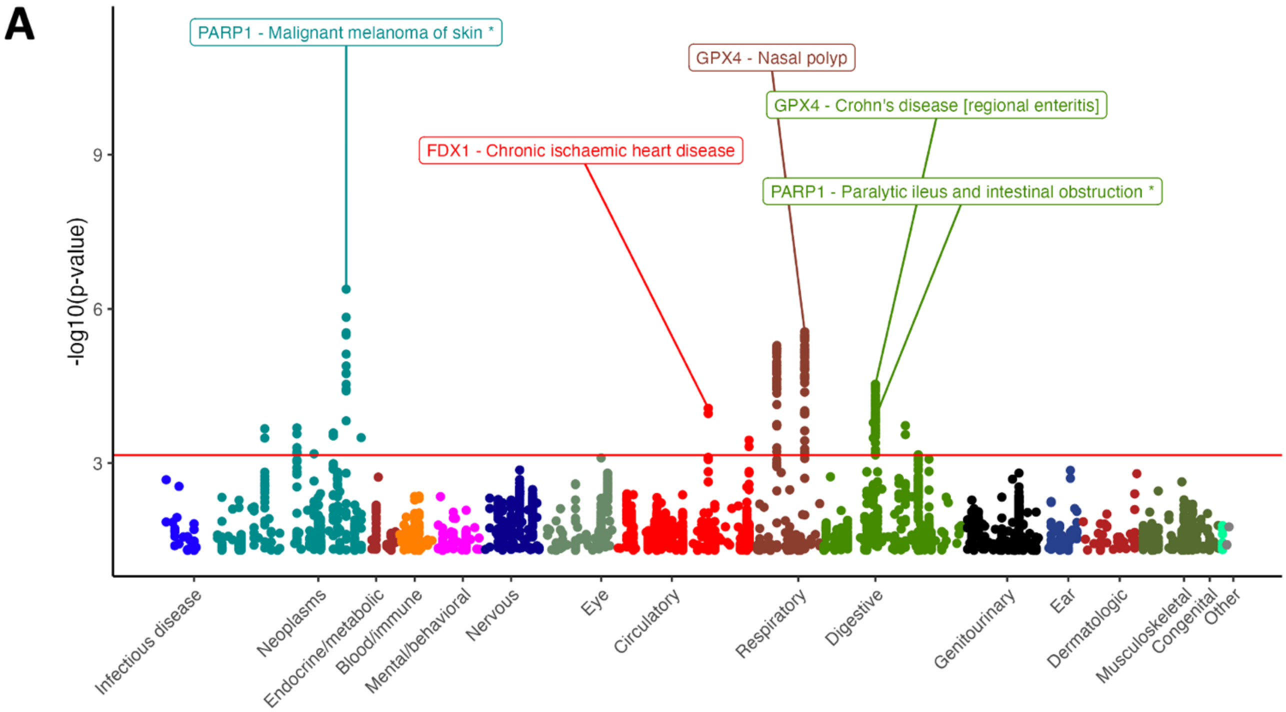


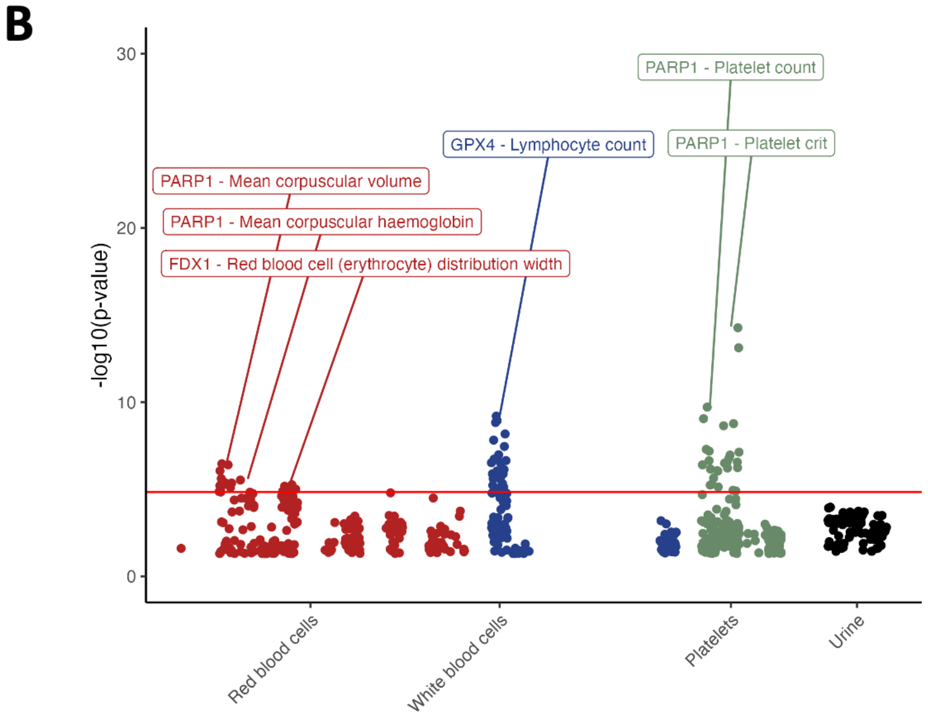


**Supplementary Figure 3: Trait associations with central regulators of ferroptosis, cuproptosis, and parthanatos pathways.**

**A** Manhattan plot illustrating top gene-trait associations by p-value and organized by trait type. Only the most significant gene-trait association in a phenotype category is labeled. Gene-trait associations with significant associations across multiple tissues (i.e. *GPX4* and Nasal polyp), are not annotated for clarity.

**B** Manhattan plot illustrating top laboratory-derived gene-trait associations by p-value and organized by trait type. Unique gene/trait associations passing Bonferroni cutoff of p=1.440092e-05 are labeled, and the red line illustrates this threshold. Gene/trait associations occurring across multiple tissues are not labeled in this plot for clarity.
